# Differential diagnostic performance of acoustic radiation force impulse imaging in small (≤20 mm) breast cancers: Is it valuable?

**DOI:** 10.1038/s41598-017-08004-y

**Published:** 2017-08-17

**Authors:** Si-Da Wang, Lei Wang, Zhi-Xian Li, Kang-Lai Wei, Xin-Hong Liao, Yuan-Yuan Chen, Xue Huang

**Affiliations:** 1 The First Affiliated Hospital of Guangxi Medical University, Department of Ultrasound Diagnosis, Nanning, 530021 Guangxi China; 2 Shandong Provincial Hospital Affliated to Shangdong University, Department of Ultrasound Diagnosis, Jinan, 250021 Shandong China; 3 The First Affiliated Hospital of Guangxi Medical University, Department of Pathological Diagnosis, Nanning, 530021 Guangxi China

## Abstract

To evaluate acoustic radiation force impulse (ARFI) inthe differential diagnosis of small (≤20 mm) solid breast lesions and identify the most efficient ARFI parameters. Conventional ultrasonography and ARFIwere performed in 120 patients with 121 small solid breast lesions. The area ratios (ARs) of the lesion on virtual touch tissue compared to B-mode were calculated. The shear wave velocity of the inner (SWVi) and boundary (SWVb) of the lesions and surrounding fatty tissue (SWVf) was measured. The ratio of SWVi to SWVf (SWVrat) was calculated. AR, SWVi, SWVb, and SWVrat were significantly larger in malignant lesions (all *P* < 0.001). A cutoff AR of 1.17 yielded the highest area under the receiver operating characteristic curveamong the various parameters (91.2% sensitivity, 85.9% specificity, 88.4% accuracy) for the differential diagnosis of small breast lesions, but this value did not significantly differ from SWVi (*P* = 0.1144). This AR cutoff indowngradingcategory 4a to category 3 would avoid 83.3% unnecessary biopsies, and improved diagnostic specificity up to 73.4% without decreasing sensitivity. AR and SWVi are efficient parameters for the differential diagnosis of small breast lesions, whichwill improve diagnostic specificity and reduce unnecessary biopsies.

## Introduction

Breast cancer is the most frequently diagnosed life-threatening cancer and the second leading cause of cancer deaths among Asian women^1, 2^. The detection rate of breast cancers has increased over time due to the development of screening strategies. However, in Asian women, who tend to have dense breast tissue, malignant masses especially small masses may not be detectable on mammography^3–5^. Furthermore, breast cancer in the advanced stage has a poor prognosis^6^. Records show that T3 breast cancer lesions are associated with a 10-year survival rate of less than 60%, while T1 lesions (≤20 mm) are associated with a 10-year survival rate of approximately 85%^7^. Hence, the early diagnosis of breast cancer is a cornerstone of successful treatment.

Ultrasonography (US) has emerged as an indispensable tool in the diagnosis of breast disease^8, 9^. In recent years, ultrasound elastography, which reflects tissue stiffness, has been used to differentiate between benign and malignant lesions^10–13^. Acoustic radiation force impulse (ARFI) imaging is a type of ultrasound elastography without external compression. In ARFI imaging, short-duration acoustic pulses are used to generate localized tissue displacement^14^, and the resulting microscale displacement, which has been shown to be proportional to the square root of tissue stiffness, is tracked using conventional B-mode imaging pulses. Depending on the interactions between the transducer and waves, ARFI imaging can be performed in two different modes: virtual touch tissue imaging (VTI), which generates a gray-scale map, and virtual touch tissue quantification (VTQ), which determines shear wave velocity (SWV) values (measured in meters per second)^15^. The stiffer the tissue, the darker the appearance and faster the SWV on VTI and VTQ, respectively^16^.

In recent years, several clinical studies have reported that ARFI imaging is useful for differentiating between benign and malignant breast lesions^14, 17, 18^. However, to our knowledge, no study has specifically assessed the value of ARFI imaging in the evaluation of small solid breast lesions. The purpose of our study was to determine the value of ARFI in the differential diagnosis of small (≤20 mm) solid breast lesions, and identify the ARFI parameter that proved to be the most efficient.

## Results

### Pathological diagnoses and BI-RADS classification

Of the 121 lesions, 64 (52.89%) were benign, and 57 (47.11%) were malignant on histopathological examination. The histopathological results are summarized in Table 1. Only two lesions (one chronic mastitis and one mucinous carcinoma) were found to contain a few cystic areas on pathological examination; the rest were all solidlesions. The most common benign and malignant lesions were fibroadenoma and invasive ductal carcinoma, respectively. The mean maximum diameter of malignant breast lesions was significantly larger than that of the benign lesions (16.02 ± 3.46 mm vs. 14.62 ± 4.13 mm, *P* = 0.046). The TNM stages of the malignant breast lesions are summarized in Table 2.

**Table 1:** Results of the histopathological examinationof small solid breast lesions.

Histology	No. of lesions (%)
Benign	64
Fibroadenoma	38 (59.4)
Adenosis	15 (23.4)
Intraductal papilloma	6 (9.4)
Mastitis, chronic or granulomatous	4 (6.3)
Fibroadenomatous hyperplasia	1 (1.5)
Malignant	57
Invasive ductal carcinoma	48 (84.2)
Ductal carcinoma *in situ*	3 (5.3)
Ductal carcinoma *in situ* with microinvasion	3 (5.3)
Invasive lobular carcinoma	2 (3.5)
Mucinous carcinoma	1 (1.7)

**Table 2:** TNM staging of small solid breast malignant lesions (n = 57).

Stage	0	I	II	III	IV
Lesions (n = 57)	6	46	5	0	0

The malignancy rates for each BI-RADS-US category were as follows: 3.6% (1/28) for category 3, 8.3% (2/24) for category 4a, 43.5% (10/23) for category 4b, 90.0% (18/20) for category 4c, and 100.0% (26/26) for category 5. With a cutoff point between category 3 and 4a, the sensitivity, specificity, accuracy, PPV, and NPV of BI-RADS-US were determined to be 98.2%, 42.2%, 60.2%, 96.4%, and 68.6%, respectively, and the AUC value was 0.944 (95% CI: 0.886, 0.977).

### VTI and VTQ

**Figure 1:**
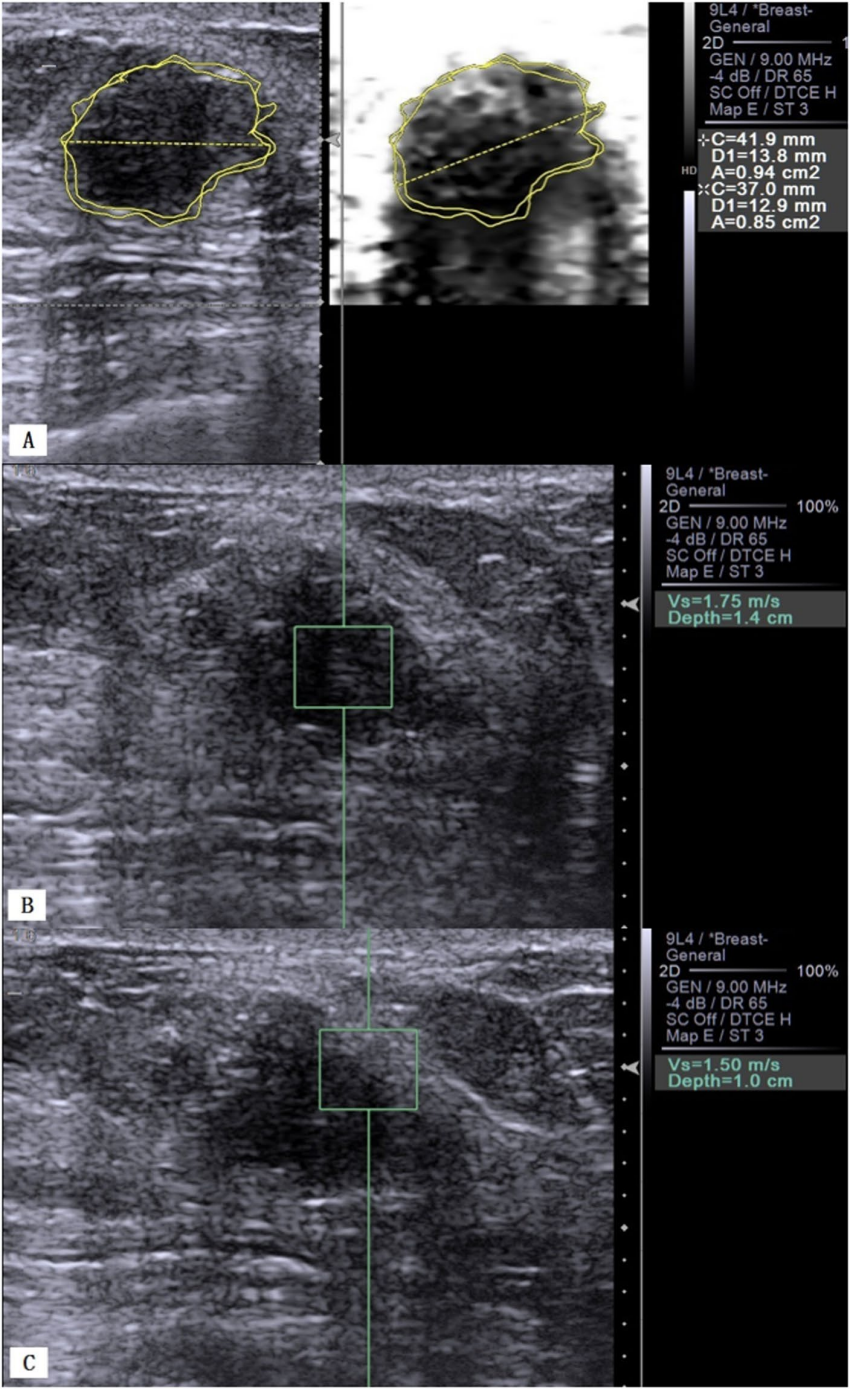
Fibroadenoma of the right breast in a 36-year-old woman. (**A**) B-mode US shows a 12.9 mm hypoechoic solid lesion with irregular margins (left); VTI shows a dark lesion with an AR = 1.11 (0.94/0.85) (right). (**B**) VTQ measures an SWV of 1.75 m/s in the lesion. (**C**) VTQ measures an SWV of 1.50 m/s in the boundary zone of the lesion.

**Figure 2:**
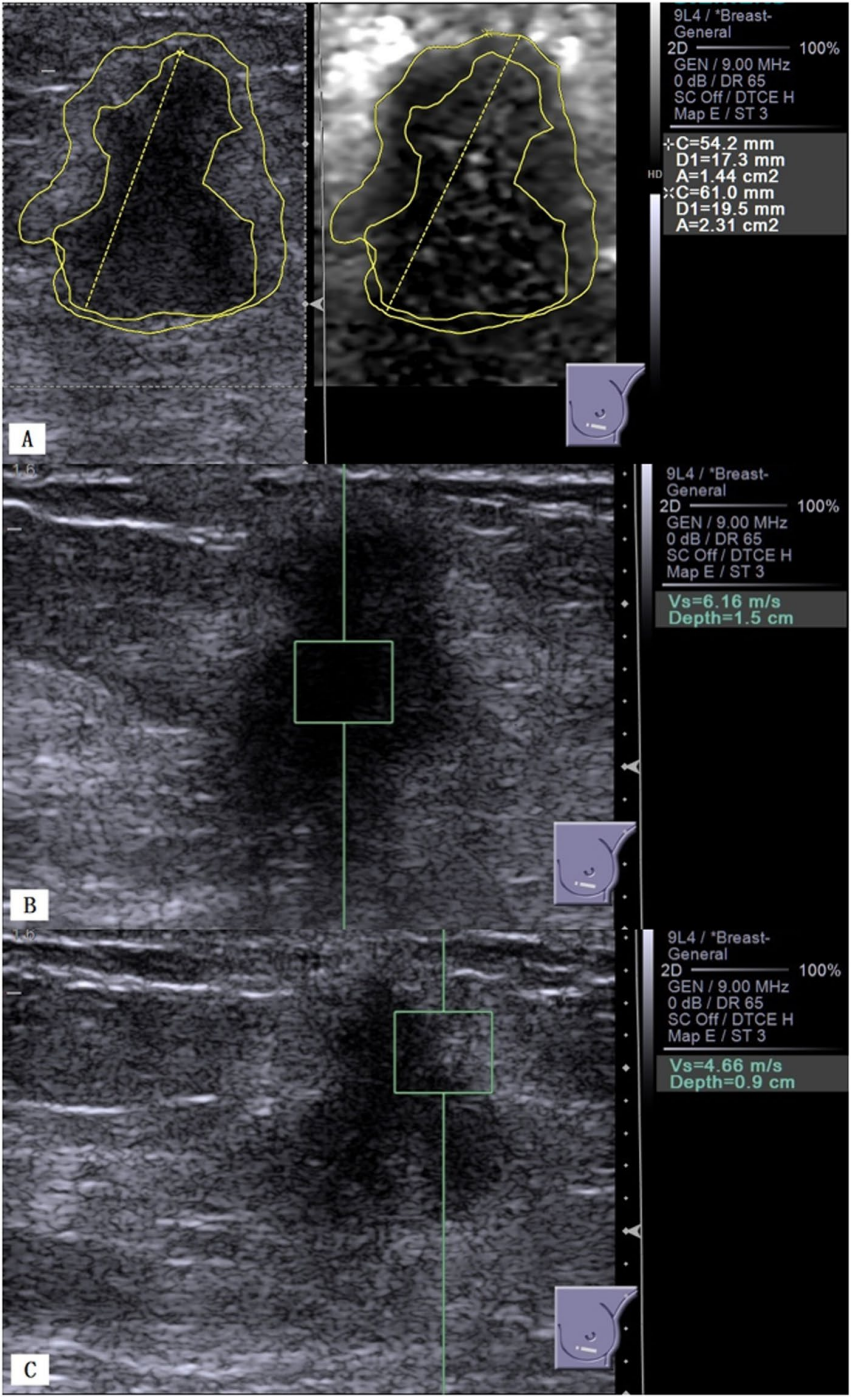
Invasive ductal carcinoma of the left breast in a 50-year-old woman. (**A)**, B-mode US shows a 19.6 mm hypoechoic solid lesion with irregular margins (left); VTI shows a dark lesion with an AR = 1.60 (2.31/1.44) (right). (**B**) VTQ measures an SWV of 6.16 m/s in the lesion. (**C**) VTQ measures an SWV of 4.66 m/s in the boundary zone of the lesion.

The mean AR of the malignant lesions was significantly larger than that of the benign lesions (1.34 ± 0.23 vs. 0.99 ± 0.18, *P* < 0.001; Figs 1 and 2). ROC curve analysis for the differentiation between benign and malignant lesions gave a cutoff AR value of 1.17, and this cutoff point yielded a sensitivity of 91.2% (52/57), specificity of 85.9% (55/57), accuracy of 88.4% (107/121), PPV of 85.2% (52/61), NPV of 91.7% (55/60), false-positive rate of 14.1% (9/64), and false-negative rate of 8.8% (5/57). The AUC was 0.921 (95% CI: 0.889, 0.974).

The malignant group showed significantly higher SWVi (4.06 ± 1.62 m/s vs. 2.15 ± 0.63 m/s), SWVb (3.42 ± 1.67 m/s vs. 1.85 ± 0.61 m/s), and SWVrat (4.33 ± 1.88 vs. 2.37 ± 0.80)values than did the benign group (all *P* < 0.001, Table 3). The diagnostic performance of the SWV quantitative parametershas been summarized in Table 4. An optimal SWVi cutoff of 3.09 m/s yielded a sensitivity of 68.4% (39/57), specificity of 93.7% (60/64), accuracy of 81.8% (99/121), PPV of 90.7% (39/43), NPV of 16.9% (60/78), false-positive rate of 6.3% (4/64), and false-negative rate of 25% (14/57). It was associated with the highest AUC value among all SWV parameters (0.851, 95% CI: 0.775, 0.909), but did not significantly differ from other SWV parameters (all *P* > 0.05).

**Table 3:** Comparison of size, AR, and SWV between the benign and malignant groups.

Characteristic	Age(years)	Size(mm)	AR	SWVi(m/s)	SWVb(m/s)	SWVf(m/s)	SWVrat
Benign (n = 64)	36.86 ± 10.49	14.62 ± 4.13	0.99 ± 0.18	2.15 ± 0.63	1.85 ± 0.61	0.93 ± 0.12	2.37 ± 0.80
Malignant (n = 57)	49.74 ± 11.17	16.02 ± 3.46	1.34 ± 0.23	4.06 ± 1.62	3.42 ± 1.67	0.97 ± 0.18	4.33 ± 1.88
*P*-value	<0.001	<0.05	<0.001	<0.001	<0.001	>0.05	<0.001

Note: abbreviations: AR, area ratio; SWV, shear wave velocity; SWVi, shear wave velocity of inner region; SWVb, shear wave velocity of boundary zone; SWVf, shear wave velocity of fatty tissue; SWVrat, ratio of SWVi to SWVf.

**Table 4:** Diagnostic performances of conventional US, ARFI parameters and US&ARFI for small solid breast lesions.

Characteristic	Cutoff	Sensitivity (%, n)	Specificity (%, n)	Accuracy (%, n)	PPV (%, n)	NPV (%, n)	AUC (95% CI)	*P*-value^a^	*P*-value^b^
BI-RADS	4a	98.2 (56/57)	42.2 (27/64)	68.6 (83/121)	60.2 (56/93)	96.4 (27/28)	0.944 (0.886, 0.977)	—	
AR	1.17	91.2 (52/57)	85.9 (55/64)	88.4 (107/121)	85.2 (52/61)	91.7 (55/60)	0.921 (0.858, 0.962)	0.477	—
SWVi (m/s)	3.09	68.4 (39/57)	93.7 (60/64)	81.8 (99/121)	90.7 (39/43)	76.9 (60/78)	0.851 (0.775, 0.909)	0.0104	0.1144
SWVb (m/s)	1.95	84.2 (48/57)	70.3 (45/64)	76.9 (93/121)	71.6 (48/67)	83.3 (45/54)	0.844 (0.767, 0.904)	0.0088	0.0371
SWVrat	3.04	71.9 (41/57)	85.9 (55/64)	79.3 (96/121)	82.0 (41/50)	77.5 (55/71)	0.823 (0.743, 0.887)	0.0014	0.0393
BI-RADS&AR	—	89.5 (51/57)	85.9 (55/64)	87.6 (106/121)	85.0 (51/60)	90.2 (55/61)	0.934 (0.874, 0.971)	0.408	
BI-RADS&SWVi	—	73.7 (42/57)	93.7 (60/64)	84.3 (102/121)	91.3 (42/46)	80.0 (60/75)	0.859 (0.784, 0.916)	0.0033	
BI-RADS&SWVb	—	84.2 (48/57)	73.4 (47/64)	78.5 (95/121)	73.8 (48/65)	83.9 (47/56)	0.874 (0.801, 0.927)	0.0415	
BI-RADS&SWVrat	—	71.9 (41/57)	87.9 (56/64)	80.2 (97/121)	83.7 (41/49)	77.8 (56/72)	0.834 (0.756, 0.896)	0.0005	

Note: abbreviations: AR, area ratio; AUC, area under the receiver operating characteristic curve; ARFI, acoustic radiation force impulse; BI-RADS, Breast Imaging Reporting and Data System; CI, confidence interval; NPV, negative predictive value; PPV, positive predictive value; SWV, shear wave velocity; SWVb, shear wave velocity of boundary zone; SWVf, shear wave velocity of fatty tissue; SWVi, shear wave velocity of inner region; SWVrat, ratio of SWVi to SWVf; US, ultrasonography. ^a^Compared to the AUC value of BI-RADS; ^b^compared to the AUC value of AR.

**Figure 3:**
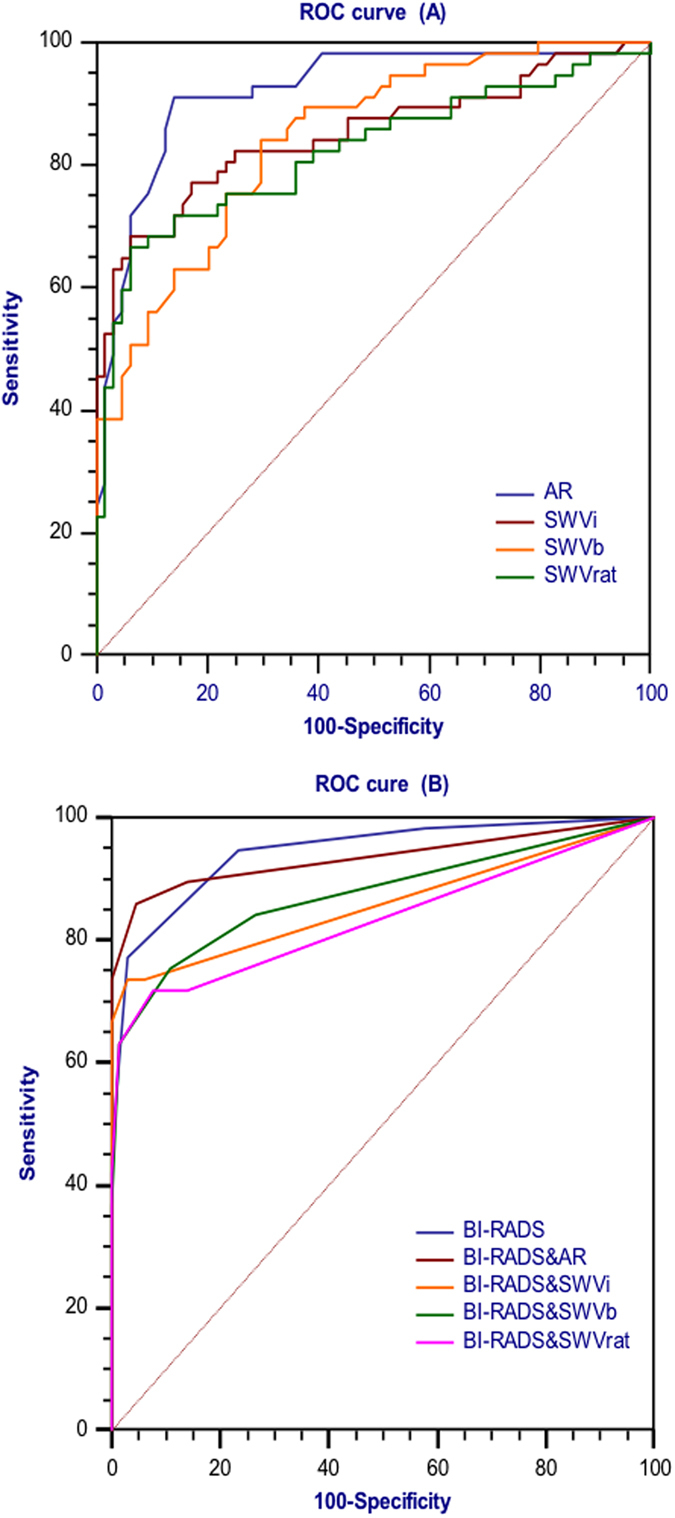
(**A**) Receiver operating characteristic (ROC) curves for the diagnostic performance of AR and each SWV parameter in differentiating benign and malignant small solid breast lesions. The area under the ROC curve for AR was significantly higher than those for SWVb and SWVrat (*P* < 0.05), but did not significantly differ from that for SWVi (*P* > 0.05). (**B**) ROC curves for the diagnostic performance of BI-RADS, BI-RADS combined with AR, and BI-RADS combined with each SWV parameter in differentiating benign and malignant small solid breast lesions. The area under the ROC curve for BI-RADS significantly differedfrom those for BI-RADS combined with each SWV parameter (*P* < 0.05), but did not differ from that for BI-RADS combined with AR (*P* > 0.05).

Among all the VTI and VTQ parameters, AR had the highest AUC value, but did not significantly differ from SWVi in its ability to differentiatebetween benign and malignant lesions (*P* = 0.1144). However, AR did significantly differ from other SWV parameters (*P* < 0.001, Fig. 3A). Comparedwith BI-RADS-US, the combination of BI-RADS-US with ARand each SWV parameter increased the diagnostic specificity from 42.2% to 73.4–93.7% and accuracy from 68.6% to 78.5–87.6%. However, the sensitivity decreased from 98.2% to 71.9–89.5%. Furthermore, the AUC value of BI-RADS-US was still the highest at 0.944 (95% CI: 0.886, 0.977), and this value significantly differedfrom the values obtained by combining BI-RADS-US with each SWV parameter (all *P* < 0.05, Fig. 3B).

### Effect of ARFI parameters on BI-RADS category 4a lesions

Since SWVi had the highest specificity, we used a SWVi of <3.09 m/s to downgrade soft BI-RADS category 4a lesions to category 3 lesions. With this criterion, all 24 lesions category 4a lesions in this study were downgraded to category 3 lesions; this would have resulted in the avoidance of 91.7% (22/24) unnecessary biopsies, but would have missed 2 breast cancer lesions. Furthermore, the malignancy rate of the category 3 lesions after the reclassification increased from 3.7% to 5.8%. When an SWVi cutoff of <2.5 m/s was used for downgrading, 20 category 4a lesions, including 2 cancers, were downgraded to category 3 lesions; this would have avoided 75.0% (18/24) of unnecessary biopsies, but further increased the malignancy rate of category 3 lesions from 3.7% to 6.3%. The most conservative cutoff of SWVi <1.8 m/s resulted in the downgrading of 41.7% (10/24) of benign category 4a lesions to category 3 lesions. This cutoffdecreased the malignancy rate of category 3 lesions from 3.7% to 2.6%, and improved the overall specificity from 42.2% (27/64) to 57.8% (37/64), with no decrease in sensitivity.

The application of an AR cutoff of <1.17 for downgrading category 4a lesions to category 3 lesionsresulted in the downgrading of 20 of 24 lesions. All 20 downgraded lesions were benign, and thus, 83.3% (20/24) of unnecessary biopsies would have been avoided. Furthermore, the malignancy rate of the category 3 lesions decreased from 3.7% to 2.1% after the reclassification. In addition, the overall specificity of BI-RADS-US improved from 42.2% (27/64) to 73.4% (47/64), with no decrease in sensitivity.

## Discussion

Non-invasive examinations such as mammography, US, and MRI can improve the rates of early diagnosis of breast cancer. However, mammography has a lower diagnostic performance in women with dense breast tissues and is a radiation hazard, while MRI is time-consuming and expensive.In contrast, USis unaffectedby the density of breast tissue, and is safe, convenient, and inexpensive.

Conventional US images can characterizelesions according to their acoustic properties, which are described using the current BI-RADS descriptors, and these in turn are used to classify the lesion into the appropriate category. Although BI-RADS has a high sensitivity, its specificity is low, especially, in the case of small lesions classified into category 4a or 4b^12, 19^. In our study, the diagnostic sensitivity of BI-RADS was 98.2%; however, its specificity was only 42.2%. This may be partially attributable to the fact that US is highly dependent on the operator’s experience, and subjective operator-related factors may affect the BI-RADS category of the lesion.

In recent years, there has been a growing interest in breast elastography, which is imaged using US. Among the different elastography techniques, ARFI is an efficient method that does not require external compression and can provide quantitative information about lesions. Elastography can enhance the contrast between malignant lesions and the background normal breast tissue^20^. Malignant lesions appear larger on VTI than on the corresponding conventional US image due to desmoplastic reaction and infiltrative growth, while benign lesions appear similar in size^21^. As expected, in our study, the AR values were significantly higher for malignant breast lesions than for benign lesions. This result is consistent with those reportedby Bai *et al*.^14^ and Meng *et al*.^21^. Malignant breast lesions are stiffer than benign lesions and normal breast tissues^16, 22^, and our data showed that all SWV parameters were significantly higher in the malignant group than in the benign group, which is consistent with previously published data^11, 16–18^.

In our study, AR had the best diagnostic performance for small solid breast lesions among all the parameters evaluated, although it did not significantly differ from the performance of SWVi. All ARFI parameters had significantly higher specificities, accuracies, and PPVs than those of BI-RADS-US (all *P* < 0.001); nevertheless, the overall diagnostic performance in small breast cancers was not improved. After combining US with ARFI parameters, the specificity increased from 42.2% to 73.4–93.7%, and accuracy increased from 68.6% to 78.5–87.6%, similar results have been reported in previous studies^12, 17, 19^. However, the sensitivity decreased from 98.2% to 71.9–89.5%, and the AUC decreased from 0.944 to 0.834–0.934. Furthermore, BI-RADS-US still had the bestdiagnostic performance; these results are similar to those reported by Kim *et al*.^23^.

Among the ARFI parameters, SWVi had the highest diagnostic specificity of 93.7%, which was much higher than that of BI-RADS-US. Of the 64 benign lesions, 4 (6.3%) were falsely positive, including 2 fibroadenomas, 1 intraductal papilloma, and 1 sclerosingadenosis, with SWVi values ranging from 3.26 m/s to 4.29 m/s. Both sclerosingadenosis and intraductalpapillomas are in the subset of lesions that appear falsely positive on elastography^23, 24^. Histopathological examination of the 2 falsely positive fibroadenomas revealed obvious hyperplasia of the interstitial fibrous tissues and squeezed components of the glandular epithelium. However, further research with larger samples is needed to identify the causes of the falsepositivity. When we applied an SWVi cutoff value of 3.09 m/s for downgrading category 4a lesions to category 3 lesions, 91.7% of category 4a lesions were reclassified as benign lesions, which would have avoided many unnecessary biopsies; however, this reclassification would also have missed 2 malignancies, including 1 ductal carcinoma *in situ* with microinvasion measuring 12.3 mm and 1 ductal carcinoma *in situ* measuring 10.4 mm. The malignancy rate of the category 3 lesions increased from 3.7% to 5.8% after the reclassification, and thus, this result was undesirable. The most conservative strategy recommended for downgrading category 4a lesions in previous studies^12, 23^ resulted in the downgrading of 41.7% of category 4a lesions, without any increase in the malignancy rate of category 3 lesions. However, the overall diagnostic specificity of BI-RADS-US only improved from 42.2% to 57.8%. When we used an AR cutoff value of 1.17 to downgrade category 4a lesions, we found that 83.3% of unnecessary biopsies would be avoided. Furthermore, the malignancy rate of the category 3 lesions decreased from 3.7% to 2.1%. In addition, the overall diagnostic specificity of BI-RADS-US improved from 42.2% to 73.4% without any loss of sensitivity. Therefore, AR might be a more suitable parameter for downgrading BI-RADS category 4a lesions to category 3 lesions.

There are several limitations to this study. First, the SWV values measured by the machine in this study were limited to a range of 0–9 m/s. SWV values exceeding the upper limit were underestimated. Second, the sample size of this study was relatively small, and the results did not represent all pathological types of small breast lesions. Further studies with a larger sample size are necessary to validate our results. Third, all US examinations were performed by two sonographers, and the BI-RADS-US classification was done by two observers in our study. However, intraobserver and interobserver variabilities were not considered. Fourth, the malignancy rate of category 3 lesions in this study (3.7%)was higher than the BI-RADS recommendations^25^. This may be because many patients enrolled in this study had been screened at the out-patient department, and few patients with category 3 small breast lesions were hospitalized.

In conclusion, the ARFI technique might be useful to increase the diagnostic specificity and accuracy for small breast cancers(≤20 mm). The number of patients requiring further MRI examination and unnecessary biopsycan be reduced. In all parameters, AR and SWVi are efficient parameters for the differential diagnosis of small breast lesions. However, AR is better than SWVi for downgrading BI-RADS category 4a lesions.

## Methods

### Patients

This prospective study was approved by the institutional review board of First Affiliated Hospital of Guangxi Medical University, and all participants signed informed consent forms. In this study, all operations were in compliance with relevant guidelines and regulations. From March 2015 to November 2015, a total of 120 women with 121 small solid breast lesions (maximum diameteron US, ≤20 mm; range, 6.0–20.0 mm) treated at the Breast Surgery Department of the First Affiliated Hospital of Guangxi Medical Universitywere enrolled in the study. The mean age of the participants was 42.9 ± 12.6 years (range, 17–76 years).None of the lesions had previously been treated with radiotherapy or chemotherapy. Conventional US and ARFI imaging were performed in all patients prior to US-guided core-needle biopsy or surgical excision, and each breast lesion was diagnosed pathologically.

### Conventional US and ARFI imaging

Conventional US and ARFI imaging were performed with a Siemens ACUSON S2000 ultrasound system (Siemens Medical Solutions, Mountain View, CA, USA) equipped with a linear array transducer (9L4, Siemens) with a bandwidth of 4–9 MHz. All US examinations were independently performed by two radiologists, who had 10 and 14 years of experience in breast US, and were well trained in ARFI. Before the US examinations, the radiologists were not informed of the patients’ clinical status. Conventional US scanning was performed, and the lesionswere described using the Breast Imaging Reporting and Data System (BI-RADS) lexicon^19^ with the following sonographic descriptors: mass shape, mass margin, mass boundary, mass orientation, echo pattern, and posterior acoustic features. All 121 lesions appeared solid on US images. The lesions were classified according to the BI-RADS criteria by means of consensus between the two examiners as follows: category 3 (probably benign), 28 lesions; category 4 (suspicious of malignancy, with a malignancy rate ranging from 2% to 94%), 67 lesions; and category 5 (highly suggestive of malignancy, with a more than 94% rate of malignancy), 26 lesions. There were no category 1 (negative) or 2 (benign) lesions. Category 4 lesions were further classified as category 4a (24 lesions), 4b (23 lesions), or 4c (20 lesions), according to the level of suspicionof malignancy (low, intermediate, and moderate, respectively) by means of consensus.

After conventional US scanning, ARFI examination was performed for each breast lesion without any compression. First, the lesion section with the largest diameter was obtained, and a region of interest (ROI) was placed on the lesion such that a sufficient quantity of the surrounding tissues was included in the ROI. The patient was asked to hold her breath, and the VTI button was pressed to obtain a satisfactory image. The area of the lesion was measured in the VTI and Bmodes, and the ratio of the former to the latter was calculated. The same operation, measurement, and calculation were done on two different sections. After obtaining three values from each section, the mean value was calculated as the area ratio (AR). Then, the radiologist switched to the VTQ model, and measured the SWV of the lesion and the normal breast tissues by using a quantification ROI (with fixed dimensions of 5 mm × 6 mm) placed on transverse and longitudinal sections. Five measurements from different regionsof each section were obtained, and the mean values of the ten measurements were calculated. The following parameters were used for analysis: mean SWV values obtained from the inner regions (SWVi), boundary zones (SWVb), and normal fatty tissues (SWVf), as well as the SWVrat, which is the ratio of the SWVi to the SWVf. The US machine that we used had an SWV limit of 0–9 m/s for local tissues (such as heterogeneous components); when the SWV was outside this range, the SWV value was displayed as “X.XX”. In this case, the VTI scan appeared dark, and the SWV value of the local tissue was recorded as 9 m/s.

### Statistical analysis

All statistical analyses were performed using SPSS software version 17.0 (SPSS, Chicago, IL, USA) and MedCalc software version 11.4.2 (MedCalc, Ostend, Belgium). Data were expressed as mean ± standard deviation. Continuous variables were compared between the benign and malignant groups by using the independent two-samples*t*-test. The diagnostic performance of BI-RADS-US and each quantitative parameter obtained using VTI and VTQ were evaluated using receiver operating characteristic (ROC) curve analysis. BI-RADS-US category 4a was considered as the cutoff value. The optimal cutoff value for each quantitative parameter obtained from the VTI and VTQ analyses was determined using the Youden index (sensitivity + specificity − 1) calculated from the ROC curve. The sensitivity, specificity, accuracy, positive predictive value (PPV), and negative predictive value (NPV) of BI-RADS-US and each quantitative parameter were obtained using the optimal cutoff values. The 95% confidence interval (CI) and the area under the ROC curve (AUC) were calculated.

In addition, by applying the optimal cutoff values of AR and SWV to downgrade BI-RADS-US category 4a lesions, we determined the impact of these parameters on the overall diagnostic performance of the BI-RADS-US by using ROC curves. All tests were two-sided, and *P* values of <0.05 were considered statistically significant.
